# Influence of Combination Antiretroviral Therapy on HIV-1 Serological Responses and Their Implications: A Systematic Review and Meta-Analysis

**DOI:** 10.3389/fimmu.2022.844023

**Published:** 2022-03-30

**Authors:** Yuanhao Liang, Hongqing Lin, Emmanuel Enoch Dzakah, Shixing Tang

**Affiliations:** ^1^ Guangdong Provincial Key Laboratory of Tropical Disease Research, Department of Epidemiology, School of Public Health, Southern Medical University, Guangzhou, China; ^2^ Department of Molecular Biology and Biotechnology, School of Biological Sciences, College of Agriculture and Natural Sciences, University of Cape Coast, Cape Coast, Ghana

**Keywords:** antiretroviral therapy, HIV-1 serostatus, HIV-1 reservoir, meta-analysis, anti-HIV-1 antibody

## Abstract

We aimed to analyze HIV-1 seroreversion caused by combination antiretroviral therapy (cART) and to explore antibody levels of anti-HIV-1 as an alternative biomarker of HIV-1 reservoir. We searched PubMed, Embase, the Cochrane Library, and Web of Science up to August 2021 for publications about the performance of HIV-1 serological assays or the association between antibody responses against HIV-1 and HIV-1 reservoirs. Potential sources of heterogeneity were explored by meta-regression analysis, including the year of publication, country, pretreatment viral load, sample size, the timing of treatment, time on cART, and principle or type of serological assay. Twenty-eight eligible studies with a total population of 1,883 were included in the meta-analysis. The pooled frequency of HIV-1 seronegativity is 38.0% (95% CI: 28.0%–49.0%) among children with vertical HIV-1 infection and cART initiation at the age of less than 6 months, while the percentage of HIV-1 seronegativity declined to 1.0% (95% CI: 0%–3.0%) when cART was initiated at the age of >6 months. For adult patients, 16.0% (95% CI: 9.0%–24.0%) of them were serologically negative when cART was initiated at acute/early infection of HIV-1, but the seronegative reaction was rarely detected when cART was started at chronic HIV-1 infection. Substantial heterogeneity was observed among the studies to estimate the frequency of HIV-1 seronegativity in the early-cART population (*I* ^2^ ≥ 70%, *p* < 0.05 and all), while mild heterogeneity existed for the deferred-cART subjects. Moreover, anti-HIV-1 antibody response positively correlates with HIV-1 reservoir size with a pooled rho of 0.43 (95% CI: 0.28–0.55), suggesting that anti-HIV antibody level may be a feasible biomarker of HIV-1 reservoir size.

## Introduction

The WHO recommends early combination antiretroviral therapy (cART) for people living with HIV-1 infection regardless of CD4+ T-cell counts and sets the 90-90-90 goals by 2030 (1). By the end of 2020, 27.5 million people have received cART worldwide (2). Waning HIV-1 antibody response, even seroreversion, and failed seroconversion have been reported in the cART-treated population, particularly among children with a vertical HIV-1 infection or those with acute HIV-1 infection and early initiation of cART (3–13). Since HIV-1 detection is mainly dependent on serological tests, negative serostatus in cART-treated individuals would affect the diagnosis and management of HIV-1 infection. In fact, a large number of studies have reported different frequencies of HIV-1 seronegativity (4–6, 11–42), indicating the importance of a comprehensive analysis of cART-induced seronegativity.

Since the first-generation HIV-1 serological assays were approved by the U.S. Food and Drug Administration (FDA) in 1985 (43), more sensitive immunoassays from 2nd to 5th generation of HIV-1 tests, and user-friendly HIV-1 rapid tests have been developed and implemented in diagnosis and point-of-care testing of HIV-1 (44). To date, anti-HIV-1 antibody and p24 antigen detected by HIV-1 serological assays remain the most widely used biomarkers for clinical screening and diagnosis of HIV-1 infection. However, quite different frequencies of HIV-1 seronegativity and even contradictory results about HIV-1 serological testing have been reported among cART-treated populations with HIV-1 infection (4–6, 41). Thus, it is obviously necessary to conduct a systematic review and meta-regression analysis about the performance of HIV-1 serological assays among cART-treated HIV-infected population.

Furthermore, cART could affect HIV-1 neutralizing antibody (NAb) response and their ability to inhibit HIV-1 infection *in vivo*, but the dynamics of HIV-1 NAb have not yet been fully evaluated in particular in the era of cART. Unlike the rapid development of anti-HIV-1 binding antibodies, specific HIV-1 NAbs are usually induced months or years post HIV-1 infection with relatively low or moderate titers (45–47). Testing of HIV-1 NAb may be affected by antiretroviral agents in plasma of cART-treated patients (48–51), and discordant results have been reported (52–54).

Although lifelong administration of cART is successful in inhibiting HIV-1 replication and in controlling virus titers undetectable, the current antiretroviral regimens cannot eradicate HIV-1 due to the existence of the HIV-1 reservoir, which is still the main barrier of HIV-1 cure. It is important to monitor HIV-1 reservoir size for assessing the efficacy of cART and predicting viral rebound post cessation of cART (55). Various PCR-based or culture-based assays have been developed for measuring HIV-1 reservoir size but are limited in clinical application due to high cost and complexity (56). It has been reported that the intensity of anti-HIV-1 antibody response correlated well with the size of the HIV-1 reservoir since maturation and maintenance of antibody response depend on continuous stimulation of HIV-1 antigens (17, 19, 27, 28, 33, 39, 57–64). However, inconsistent results existed (21, 23, 32, 38). In this systematic review, we adapted a meta-analysis to analyze HIV-1 seroreversion caused by cART and to explore the possibility of anti-HIV-1 levels as an alternative biomarker for HIV-1 reservoir size.

## Methods

### Search Strategy and Selection Criteria

PubMed, Embase, the Cochrane Library, and Web of Science up to August 2021 was searched for the studies about the performance of HIV-1 serological assays in cART-treated population by using the combination of terms including HIV/AIDS (“HIV” [MeSH]), antiretroviral therapy (anti-retroviral agents/therapeutic use [MeSH Terms]), performance characteristics (sensitivity and specificity [MeSH Terms]), and serologic test (serology [MeSH Terms]). Details of the search strategy for the aforementioned databases are illustrated in the **Supplementary Materials**. The reference lists of all the included studies as well as the relevant review articles were also screened to identify other related studies. In this study, the frequency of seronegativity, dynamics of HIV-1 Nab, and the association between anti-HIV titers and HIV-1 reservoir size among cART-treated patients were focused on. This meta-analysis was conducted following the guidelines of Preferred Reporting Items for Systematic Reviews and Meta-Analyses (PRISMA) (Checklist S1, see **Supplementary Materials**) (65).

The included studies met the following criteria: 1) the study subjects were infected with HIV-1 and treated by cART; 2) the data were available for determining the frequency of seronegativity; and 3) serostatus was irrelevant to the influence of maternal antibodies in vertically infected children and the window period prior to seroconversion. The following reports were excluded: 1) if they were reviews, editorials, opinions, case reports, or animal studies; 2) if the number of cART-treated and cART-naive patients was not reported separately and could not be obtained from the authors; 3) if only overall estimates of the frequency of seronegativity were reported, but the timing of treatment is not available; 4) if no sufficient data for establishing the frequency of seronegativity in cART-treated children or adults, respectively.

### Data Extraction

Two authors (YL and HL) independently extracted the following information, i.e., the first author, year of publication, country, study subjects, sample size, pretreatment viral load (VL), median VL when the serological test was conducted, median months from HIV-1 diagnosis to cART initiation, median months on cART, principle or type of serological assays, and the corresponding frequency of seronegativity of each eligible study. Any disagreement between the two authors was resolved by discussing with the corresponding author ST to reach a consensus.

### Statistical Analysis

In order to decrease the effect of studies with extremely low frequency on the overall estimate, the data were transformed with the Freeman–Tukey double arcsine function before pooling the prevalence (66). The Wilson method (67) was used to calculate the 95% CIs around these estimates since the asymptotic method produces intervals that may extend below zero (68). Furthermore, both Cochran’s *Q* (reported as χ^2^ value and *p*-value) and the *I* ^2^ statistic were applied to estimate the inter-study heterogeneity. A *p* < 0.05 from Cochrane’s chi-square (χ^2^) test or *I* ^2^ statistic value >75% indicated substantial heterogeneity (69, 70). In our study, HIV-infected children and adults were separately analyzed because an infant’s immune response is different from that of an adult. In general, early HIV-1 infection refers to the initial 6-month time period after HIV-1 acquisition (71). Therefore, the cutoff value of 6 months was used in this study to group the early-cART and deferred-cART population. The study populations are then categorized into an early-cART group (≤6 months between HIV-1 diagnosis and cART initiation) and a deferred-cART group (>6 months) based on the timing of cART initiation. In consideration of different properties and testing principles of HIV-1 serological assays (44), the pooled frequency of seronegativity was estimated in the cART-treated population according to the test principles. Definition and determination of developed and developing countries are based on the criteria of World Economic Situation and Prospects 2022 published by the United Nations. When the frequency of seronegativity was evaluated by more than two serological assays with the same testing principles, the pooled estimates were adjusted for intra-study or within-study correlation (72). In situations with high inter-studies heterogeneity, a random-effects model was used; otherwise, a fixed-effect model was adapted (70). The potential sources of heterogeneity were further investigated through meta-regression analysis, including variables of the year of publication, sample size, country, pretreatment VL, the timing of treatment, months on cART, and principle or type of serological assay. Publication bias was assessed by funnel plot as well as Egger’s and Begg’s tests (73, 74). All the analyses were done in R software (version 3.5.2, R Foundation for Statistical Computing) by using the Package “meta.” Statistical tests were two-sided, and *p* < 0.05 was considered statistically significant.

## Results

### Characteristics of the Studies on the Frequency of HIV-1 Seronegativity

**Figure 1:**
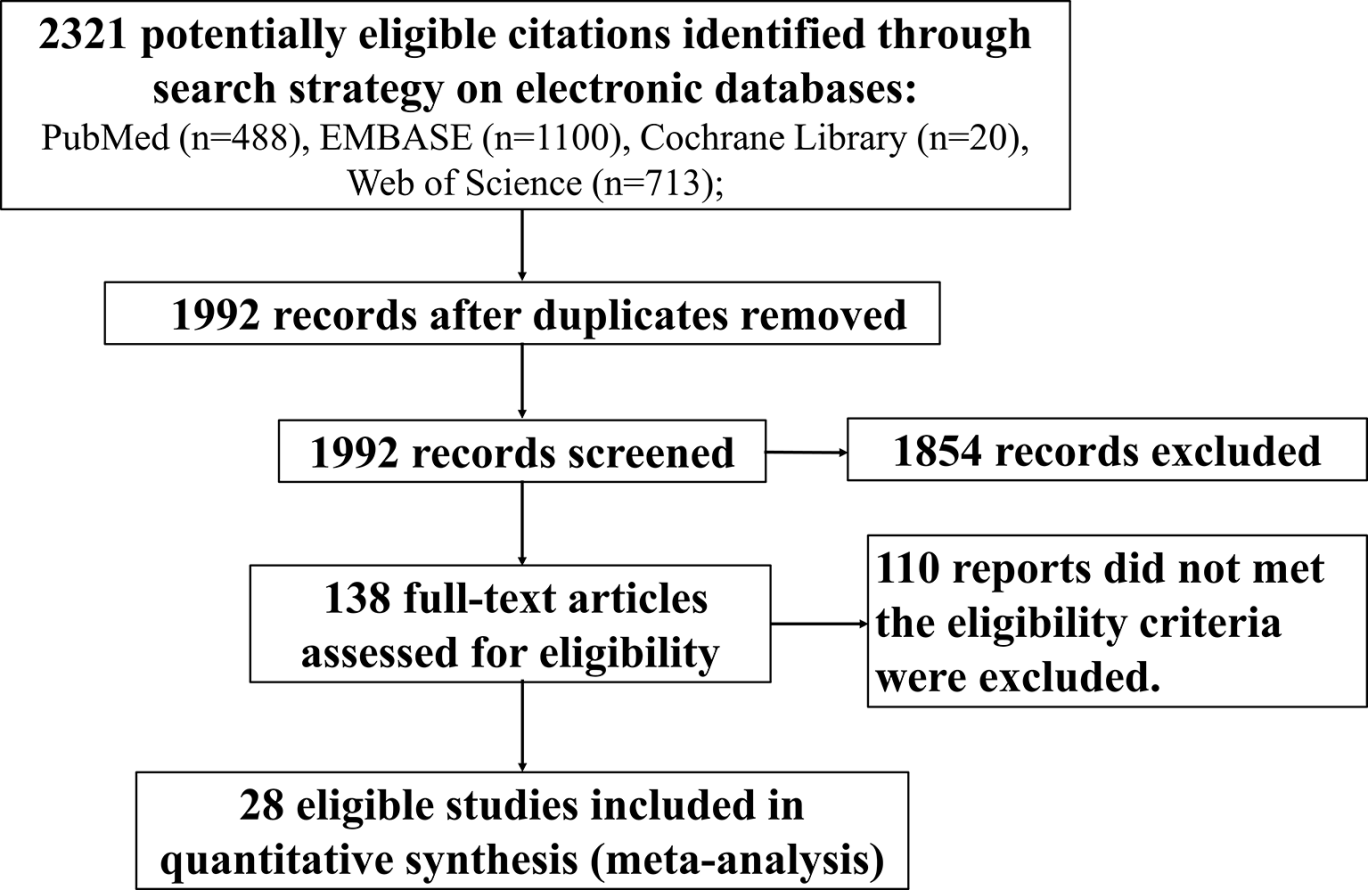
Flowchart depicting the systematic search conducted to identify eligible studies that reported frequency of HIV-1 seronegativity.

Our searches returned a total of 2,321 records from 28 studies (4, 14–29, 31–40, 75). The median sample size is 41 (interquartile range (IQR): 16–101). A total of 1,883 subjects met the eligibility criteria and were included in the meta-analysis (**Figure 1**). Eleven studies (N = 565) were conducted in the United States, 5 (N = 376) in Thailand, 3 (N = 369) in South Africa, 2 (N = 75) in Italy, and one each in Zimbabwe (N = 129), Mali (N = 97), China (N = 73), Canada (N = 69), France (N = 44), Spain (N = 14), and the United Kingdom (N = 10). There were 20 surveys with a median sample size of 29 (IQR: 13–107) to evaluate the serostatus in cART-treated vertically infected children of ≥16 months old (4, 14–29, 31–33), while the frequency of seronegativity in the cART-treated adult population was reported in 8 investigations with a median sample size of 80 (IQR: 36–101) (34–40, 75).

Among the 20 selected studies that focus on cART-treated children (**Table 1**), the frequency of seronegativity was reported in 11 studies in which early-treated children were included with a median age of 2.2 months (IQR: 1.7–2.7, N = 583) when cART started (4, 14–16, 20–22, 26, 29, 32, 33) and in 4 studies that deferred-treated children were included with a median age of 55.7 months (IQR: 37.4–86.4, N = 587) (18, 23–25). In addition, 5 studies included both early-treated and deferred-treated children and separately reported the frequency of seronegativity in the two groups (17, 19, 27, 28, 31). Of the 8 studies that investigated the serostatus in cART-treated adults (**Table 2**), 4 (34, 36, 38, 75) and 3 (35, 39, 40) studies recruited early-cART (N = 366) and deferred-cART-treated patients (N = 275), respectively. Only one study covered both early-cART (N = 9) and deferred-cART (N = 10) subjects but analyzed the frequency of seronegativity separately (37).

**Table 1:** Estimated frequency of HIV-1 seronegativity at or after 16 months of age in cART-treated vertically HIV-1-infected children.

Study ID (year)	Country	Sample size	Median VL^§^ and age at cART initiation (months)	Median time on cART (months)	Viral load at antibody test	Serological test^†^	Seronegative frequency
Luzuriaga et al. **(2000)** (4)	USA	17	5.3; 1.9	14.1	<50 copies/ml	**2nd-G EIA**	16/17 (94.1%)
Persaud et al. **(2007)** (14)	USA	12	5.8; 1.9	50.8	<400 copies/ml	**2nd-G EIA**	8/12 (66.7%)
						**Western blotting**	3/12 (25.0%)
Zanchetta et al. **(2008)** (15)	Italy	6	6; 2.8	52.5	<50 copies/ml	**3rd-G EIA**	4/6 (66.7%)
Ananworanich et al. **(2014)** (16)	Thailand	15	NA/4.0	72.7	<50 copies/ml	**4th-G EIA**	7/15 (46.7%)
Luzuriaga et al. **(2014)** (17)	USA	4	NA/2.4	203.2	<2 copies/ml	**3rd-G EIA**	3/4 (75.5%)
		4	NA/157.0	116.8	<20 copies/ml	**Western blotting**	2/4 (50.0%)
						**Western blotting**	0/4
Merchant et al. **(2014)** (18)	USA	27	NA/70.7	126.0	<50 copies/ml	**2nd-G rapid test**	4/27 (14.8%)
						**3rd-G rapid test**	0/27
						Uni-Gold™ Recombigen^®^ HIV	2/27 (7.4%)
						OraQuick Advance HIV-1/2Clearview Complete HIV ½	2/17 (11.8%)
						Clearview HIV 1/2 Stat-Pak	2/17 (11.8%)
Persaud et al. **(2014)** (19)	USA	14	5.6; 2.4	150.9	<400 copies/ml	**3rd-G EIA**	5/14 (35.7%)
		130	4.8; 32.9	124.1		**Western blotting**	2/14 (14.3%)
						**3rd-G EIA**	2/130 (1.54%)
						**Western blotting**	0/130
Kuhn et al. **(2014)** (20)	South Africa	165	4.8; 4.0	50.6	<50 copies/ml	**3rd-G EIA**	28/165 (17.0%)
		53	4.8; 11	59.6			0/53
Martínez-Bonet et al. **(2015)** (21)	Spain	14	5.6; 1.2	91.3	≤200 copies/ml	**3rd-G EIA**	4/14 (28.6%)
Payne et al. **(2016)** (22)	South African	107	5.9; 1.6	19.6	More than 80% <400 copies/ml	**3rd-G EIA**	54/101 (53.5%)
		67	5.9; 5.4	15.9		**4th-G EIA**	49/107 (45.8%)
						**3rd-G EIA**	8/67 (11.9%)
						**4th-G EIA**	8/67 (11.9%)
Brice et al. **(2017)** (23)	Mali	97	NA; 40.2	65.7	<50 copies/ml	**4th-G EIA**	8/97 (8.3%)
Foster et al. **(2017)** (24)	UK	10	5.3; 97.3	92.5	<50 copies/ml	**4th-G EIA**	0/10
Olaru et al. **(2017)** (25)	Zimbabwe	129	NA; 69.4	76.7	65.1% <1,000 copies/ml	**3rd-G rapid test**	1/129 (0.8%)
Rainwater-Lovett et al. **(2017)** (26)	USA	11	6; 2.1	133.8	<50 copies/ml	**Western blotting**	5/11 (45.5%)
Rocca et al. **(2019)** (27)	Italy	27	NA; 2.2	104.6	<50 copies/ml	**4th-G EIA**	6/27 (22.2%)
		42	NA; 42	165.5			0/42
Bitnun et al. **(2019)** (28)	Canada	10	5; 2.4	120.5	<50 copies/ml	**4th-G EIA**	3/10 (30.0%)
		30	5.7; 38.9	152.1		**4th-G EIA**	0/30
		29	5.7; 82.7	124.1		**4th-G EIA**	0/29
Puthanakit et al. **(2020)** (29)	Thailand	25	3.9; 2	36.5	88% <20 copies/ml	**2nd-G EIA**	6/25 (24.0%)
						**3rd-G EIA**	19/25 (76.0%)
						**4th-G EIA**	16/25 (64.0%)
						**Western blotting**	0/25
Wirotpaisankul et al. **(2020)** (31)	Thailand	21	NA; <6	NA	<50 copies/ml	**4th-G EIA**	7/21 (33.3%)
		89	NA; >6				2/89 (2.3%)
Ajibola et al. **(2021)** (32)	USA	38	4.1; 0.03	19.5	74% <40 copies/ml	**3rd-G EIA**	21/38 (55.3%)
Veldsman et al. **(2021)** (33)	South Africa	30	3.8; 0	24.3	<10 copies/ml	**4th-G EIA**	14/30 (46.7%)
**Timing of cART initiation is unavailable**							
Garcia-Prats et al. **(2012)** (30)	Lesotho	100	NA; NA	NA	NA	**3rd-G rapid test**	20/100 (20.0%)

NA, not available; cART, combination antiretroviral therapy.

^§^Median pretreatment HIV-1 viral load (VL), log10 copies/ml.

^†^2nd-G EIA, second-generation enzyme immunoassay; 3rd-G EIA, third-generation enzyme immunoassay; 4th-G EIA, fourth-generation enzyme immunoassay; 2nd-G rapid test, rapid test based on second-generation principles; 3rd-G rapid test, rapid test based on third-generation principles.

**Table 2:** Estimated frequency of HIV-1 seronegativity in cART-treated adult patients.

Study ID (year)	Country	Sample size	Median VL^§^ and months between diagnosis and cART initiation	Median time on cART (months)	Viral load at antibody test	Serological test^†^	Seronegative frequency
Hare et al. **(2006)** (75)	USA	87	4.6; 0.3	11.2	<500 copies/ml	**2nd-G EIA**	1/87 (1.1%)
						Vironostika HIV-1 Microelisa System	3/87 (3.4%)
						Genetic Systems HIV-1/HIV-2 Peptide EIA	2/87 (2.3%)
						**Western blotting**	15/87 (17.2%)
						Cambridge HIV-1 WB	
						Chiron HIV-1/HIV-2 RIBA	
Manak et al. **(2014)** (34)	Thailand	33	5.6; <6	2.8	NA	**3rd-G EIA**	7/33 (21.0%)
						**3rd-G rapid test**	23/33 (70.0%)
						**4th-G EIA**	7/33 (21.0%)
						**Western blotting**	9/33 (27.0%)
Piwowar-Manning et al. **(2014)** (35)	USA	101(MSM)	NA; 77.9	19.5	<50 copies/ml	**2nd-G rapid test**	0/100
						**3rd-G rapid test**	1/101 (0.8%)
						OraQuick Advance HIV-1/2	1/101 (0.8%)
						Uni-Gold™ Recombigen^®^ HIV	0/99
						**3rd-G EIA**	0/99
						**4th-G EIA**	0/99
						**Western blotting**	
de Souza et al. **(2016)** (36)	Thailand	202 (93% MSM)	5.8; 0.6	2.8	<50 copies/ml	**2nd-G EIA**	60/202 (29.7%)
				5.6		**3rd-G EIA**	6/202 (3.0%)
						**4th-G EIA**	32/202 (16.0%)
						**Western blotting**	22/187 (11.76%)
						**2nd-G EIA**	62/188 (33.0%)
						**3rd-G EIA**	8/188 (4.0%)
						**4th-G EIA**	32/188 (17.0%)
						**Western blotting**	21/187 (11.2%)
Burbelo et al. **(2018)** (37)	USA	9	4.2; <4.9	NA	<50 copies/ml	**2nd-G EIA**	2/9 (22.2%)
		10	>6	152.1		**2nd-G EIA**	0/10
Stefic et al. **(2018)** (38)	French	44	5.3; 1.4	84.2	more than 90% <50 copies/ml	**2nd-G rapid test**	4/44 (9.1%)
						**3rd-G rapid test**	3/44 (6.8%)
						VIKIA HIV1/2	13/44 (29.6%)
						Autotest VIH	0/44
						**4th-G EIA**	
Keating et al. **(2019)** (39)	USA	101	NA; >6	85.2	<50 copies/ml	**3rd-G EIA**	0/101
Liang et al. **(2020)** (40)	China	73	4.3; 34.7	24.3	<20 copies/ml	**2nd-G EIA**	0/73
**Timing of cART initiation is unavailable**							
O’Connell et al. **(2003)** (5)	USA	51	NA; NA	NA	NA	**3rd-G rapid test**	4/51 (7.8%)
O’Connell et al. **(2006)** (6)	USA	153	NA; NA	54.8	NA	**3rd-G rapid test**	0/153
Delaney et al. **(2012)** (41)	USA	386	NA; NA	NA	NA	**2nd-G rapid test**	0/383
						**3rd-G rapid test**	4/383 (1.0%)
						Clearview COMPLETE HIV-1/2	6/383 (1.6%)
						Clearview HIV-1/2 STAT-PAK	0/258
						OraQuick Advance HIV-1/2	0/376
						Multispot HIV-1/HIV-2	0/384
						Uni-Gold Recombigen HIV-1	
Fogel et al. **(2017)** (42)	Malawi	207	5.6; NA	60.8	<400 copies/ml	**3rd-G rapid test**	1/207 (0.5%)
						OraQuick Advance HIV-1/2	1/207 (0.5%)
						Uni-Gold Recombigen HIV-1	

NA, not available; MSM, men who have sex with men; cART, combination antiretroviral therapy.

^§^Median pretreatment HIV-1 viral load (VL), log10 copies/ml.

^†^2nd-G EIA, second-generation enzyme immunoassay; 3rd-G EIA, third-generation enzyme immunoassay; 4th-G EIA, fourth-generation enzyme immunoassay; 2nd-G rapid test, rapid test based on second-generation principles; 3rd-G rapid test, rapid test based on third-generation principles.

### Frequency of HIV-1 Seronegativity in Combination Antiretroviral Therapy-Treated Children

**Figure 2:**
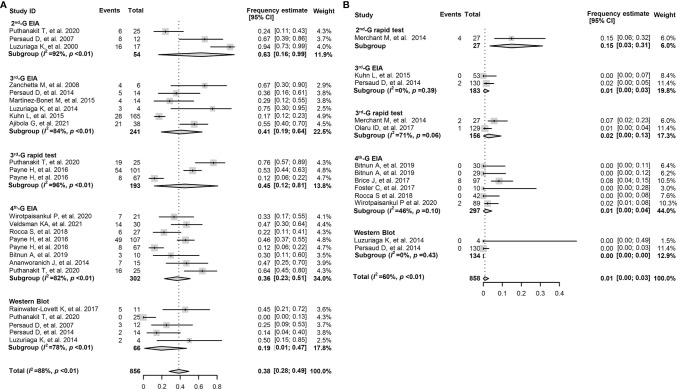
Forest plots of the pooled frequency of HIV-1 seronegativity in HIV-infected children according to the principles of HIV-1 serological assays. **(A)** The pooled frequency of HIV-1 seronegativity in vertically HIV-infected children of ≥16 months old and cART started at the age of <6 months. **(B)** The pooled frequency of HIV-1 seronegativity in vertically HIV-infected children of ≥16 months old and cART started at the age of >6 months. 2nd-G EIA, second-generation enzyme immunoassay; 3rd-G EIA, third-generation enzyme immunoassay; 4th-G EIA, fourth-generation enzyme immunoassay; 2nd-G rapid test, rapid test based on second-generation principles; 3rd-G rapid test, rapid test based on third-generation principles; cART, combination antiretroviral therapy.

The overall pooled frequency of HIV-1 seronegativity was 38.0% (95% CI: 28.0%–49.0%) among early-treated children, while the frequency declined to 1.0% (0%–3.0%) for the deferred-treated children (**Table 3**). Moreover, the frequency of HIV-1 seronegativity detected by various serological assays was significantly different. For the children who initiated cART at the age of <6 months, the pooled frequency of seronegativity was 63.0% (16.0%–99.0%) for the 2nd-G enzyme immunoassay (EIA) or 2nd-G rapid tests, 41.0% (19.0%–64.0%) for the 3rd-G EIA, 45.0% (12.0%–81.0%) for the 3rd-G rapid tests, 36.0% (23.0%–51.0%) for the 4th-G EIA, and 19.0% (1.0%–47.0%) for Western blotting analysis (**Figure 2A**). However, a relatively low frequency of seronegativity was observed in the children when cART started at the age of >6 months. The frequency of seronegativity was 15.0% (3.0%–31.0%) for the 2nd-G EIA or 2nd-G rapid tests, 1.0% (0%–3.0%) for the 3rd-G EIA, 1.0% (0%–13.0%) for the 3rd-G rapid tests, 1.0% (0%–4.0%) for the 4th-G EIA, and 0.0% for Western blotting testing (**Figure 2B**).

**Table 3:** The pooled frequency of seronegativity (95% CI) for HIV-1 serological assays in cART-treated population stratified by detection principles and timing of antiretroviral therapy started.

Serological assay classified by principles	Median age at cART initiation (children)		Median interval between diagnosis and cART initiation (adults)	
	≤6 months	>6 months	≤6 months	>6 months
**Second generation^†^**	63.0%	15.0%	14.0%	0.0%
	(16.0%–99.0%)	(3.0%–31.0%)	(1.0%–37.0%)	(0%–0.0%)
**Third generation^§^**	43.0%^§^	1.0%^§^	25.0%^§^	0.0%^§^
3rd-G EIA	(25.0%–62.0%)	(0%–3.0%)	(3.0%–57.0%)	(0%–1.0%)
3rd-G rapid test	41.0%	1.0%	10.0%	0.0%
	(19.0%–64.0%)	(0%–3.0%)	(0%–57.0%)	(0%–1.0%)
	45.0%	1.0%	43.0%	0.0%
	(12.0%–81.0%)	(0%–13.0%)	(3.0%–90.0%)	(0%–4.0%)
**Fourth generation EIA**	36.0%	1.0%	10.0%	0.0%
	(23.0%–51.0%)	(0%–4.0%)	(0%–28.0%)	(0%–2.0%)
**Western blotting**	19.0%	0	13.0%	0.0%
	(1.0%–47.0%)	(0%–0.0%)	(6.0%–22.0%)	(0%–2.0%)
**Total**	38.0%	1.0%	16.0%	0.0%
	(28.0%–49.0%)	(0%–3.0%)	(9.0%–24.0%)	(0%–0.0%)

cART, combination antiretroviral therapy; EIA, enzyme immunoassay.

^†^The frequency of seronegativity of only three 2nd-G rapid tests has been reported; thus, the 2nd-G rapid test and 2nd-G EIA have not been stratified.

^§^The pooled frequency of seronegativity of serological assays based on the third-generation, including 3rd-G rapid test and 3rd-G EIA.

### Frequency of HIV-1 Seronegativity in Combination Antiretroviral Therapy-Treated Adults

**Figure 3:**
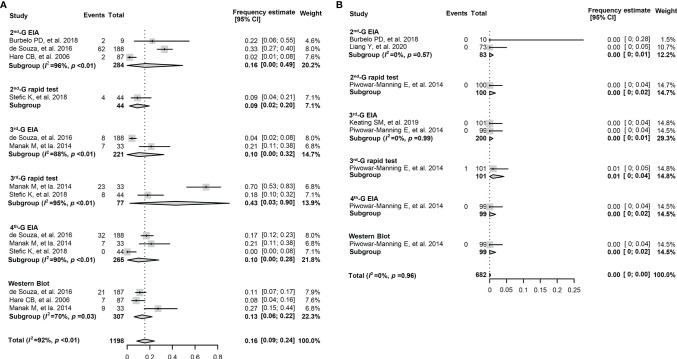
Forest plots of the pooled frequency of HIV-1 seronegativity in HIV-infected adults according to the principles of HIV serological assays. **(A)** The pooled frequency of HIV-1 seronegativity in early-treated (<6 months post diagnosis) adult patients. **(B)** The pooled frequency of HIV-1 seronegativity in deferred-treated (>6 months post diagnosis) adult patients. 2nd-G EIA, second-generation enzyme immunoassay; 3rd-G EIA, third-generation enzyme immunoassay; 4th-G EIA, fourth-generation enzyme immunoassay; 2nd-G rapid test, rapid test based on second-generation principles; 3rd-G rapid test, rapid test based on third-generation principles.

The overall pooled frequency of HIV-1 seronegativity was 16.0% (9.0%–24.0%) in the early cART-treated adult population. In contrast, only a few serologically negative cases were reported when cART started >6 months after HIV-1 diagnosis (**Table 3**). Furthermore, for the early cART-treated adult patients, the pooled frequency of seronegativity was 14.0% (1.0%–37.0%) for the 2nd-G EIA or 2nd-G rapid tests, 10.0% (0%–57.0%) for the 3rd-G EIA, 43.0% (3.0%–90.0%) for the 3rd-G rapid tests, 10.0% (0%–28.0%) for the 4th-G EIA, and 13.0% (6.0%–22.0%) for Western blotting testing (**Figure 3A**). No seronegative cases were reported in the deferred cART-treated group regardless of the serological assays used (**Figure 3B**). Due to the higher frequency of seronegativity detected by HIV-1 rapid tests, we repeated the meta-analysis by excluding the data from the 2nd and 3rd-generation rapid tests and observed similar differences of seronegativity frequency detected by different serological assays as those aforementioned (**Table 4**). Of note, the 2nd-generation EIA appeared to yield higher seronegativity frequency than other assays in both early-treated children (63.0%, 95% CI: 16.0%–99.0%) and adult patient (16.0%, 95% CI: 0%–49.0%, **Table 4**).

**Table 4:** The pooled frequency of seronegativity (95% CI) for HIV-1 serological EIA assays in cART-treated population stratified by detection principles and timing of antiretroviral therapy started.

Serological assay classified by principles^†^	Median age at cART initiation (Children)		Median interval between diagnosis and cART initiation (Adults)	
	≤6 months	>6 months	≤6 months	>6 months
**Second-generation EIA**	63.0%	NA	16.0%	0.0%
	(16.0%–99.0%)		(0%–49.0%)	(0%–1.0%)
**Third-generation EIA**	41.0%	1.0%	10.0%	0.0%
	(19.0%–64.0%)	(0%–3.0%)	(0%–32.0%)	(0%–1.0%)
**Fourth-generation EIA**	36.0%	1.0%	10.0%	0.0%
	(23.0%–51.0%)	(0%–4.0%)	(0%–28.0%)	(0%–2.0%)
**Western blotting**	19.0%	0	13.0%	0.0%
	(1.0%–47.0%)	(0%–0.0%)	(6.0%–22.0%)	(0%–2.0%)
**Total**	37.0%	1.0%	12.0%	0.0%
	(27.0%–49.0%)	(0%–2.0%)	(6.0%–20.0%)	(0%–0.0%)

NA, not available; EIA, enzyme immunoassay; cART, combination antiretroviral therapy.

^†^Both the second- and third-generation rapid tests were excluded.

### Factors Associated With the Frequency of HIV-1 Seronegativity

**Figure 4:**
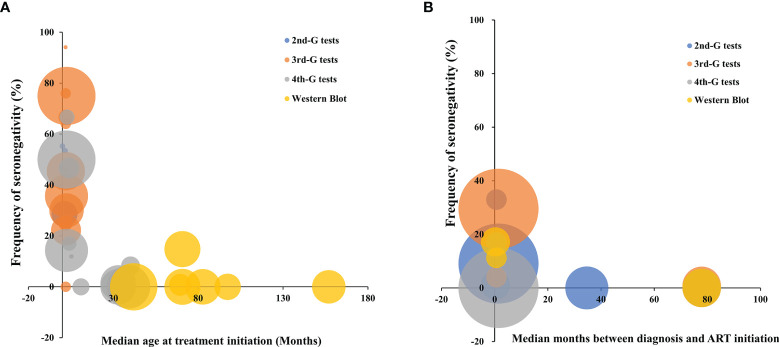
The distribution of frequency of HIV-1 seronegativity in cART-treated vertically HIV-infected children **(A)** and adult patients **(B)** according to the time of cART initiation and the principles of HIV-1 serological assays. Bubble size is proportional to the duration of cART. 2nd-G test, HIV-1 serological test based on second-generation principle; 3rd-G test, HIV-1 serological test based on third-generation principle; 4th-G test, HIV-1 serological test based on fourth-generation principle; cART, combination antiretroviral therapy.

Substantial heterogeneity was observed across the subgroups in the children with early cART (*I* ^2^ ranges from 78% to 92%, *p* < 0.01) and early cART-treated adults (*I* ^2^ ranges from 70% to 96%, *p* < 0.05). Therefore, we explored the potential sources of heterogeneity through multivariate meta-regression analysis. For cART-treated children, after other potential confounders were adjusted, only the timing of cART initiation remained significant, while deferred treatment was significantly associated with a lower frequency of seronegativity (β = −0.590, *p* < 0.001, **Table 5**). The multivariate analysis was not conducted in the cART-treated adult population since there were only 8 studies included. It is believed that meta-regression analysis is generally appropriate for the analysis of more than 10 studies. Taken together, the timing of anti-retroviral treatment including the age for the cART-treated children played a critical role in determining the serostatus of HIV-infected subjects (**Figure 4**). The frequency of HIV-1 seronegativity was inversely correlated with the time interval from HIV-1 diagnosis or infection to cART initiation for HIV-infected adults.

**Table 5:** Multivariate meta-regression for the frequency of HIV-1 seronegativity in cART-treated children.

Characteristic	Meta-regression coefficient [95% CI]	*p*-Value
**Year of publication**	−0.030 [−0.063 to 0.004]	0.086
**Sample size, continuous**	−0.001 [−0.003 to 0.002]	0.886
**Where the study was conducted (developing vs. developed countries)**	−0.269 [−0.594 to 0.056]	0.104
**Pretreatment HIV-1 viral load≥ (log10 copies/**ml**)**	−0.165 [−0.332 to 0.003]	0.054
**Median age at cART initiation (>6 vs. <6 months)**	−0.590 [−0.864 to (−0.315)]	<0.001
**Median time on cART (months)**	−0.001 [−0.004 to 0.003]	0.811
**Serological assays**		
3rd-G EIA vs. 2nd-G tests	−0.039 [−0.422 to 0.344]	0.842
3rd-G rapid test vs. 2nd-G tests	0.290 [−0.187 to 0.767]	0.233
4th-G EIA vs. 2nd-G tests	0.147 [−0.288 to 0.581]	0.508
Western blotting vs. 2nd-G tests	−0.246 [−0.641 to 0.150]	0.224

EIA, enzyme immunoassay; cART, combination antiretroviral therapy.

### Publication Bias

**Figure 5:**
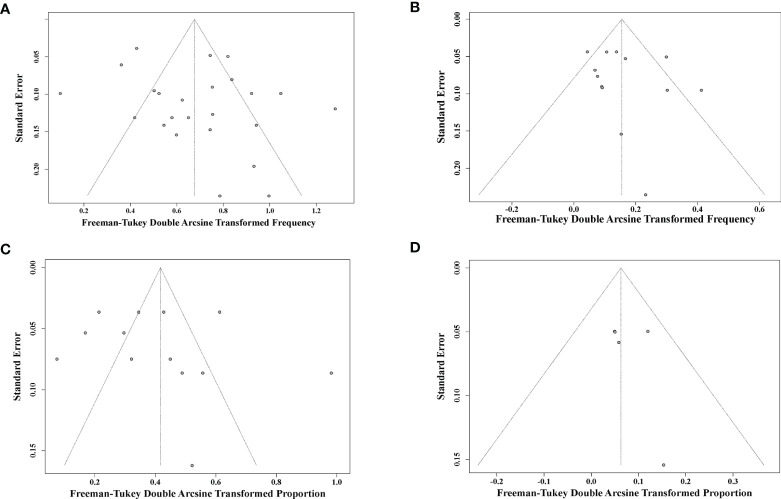
Funnel plot shows publication bias of the identified surveys for early-cART children **(A)**, deferred-cART children **(B)**, early-cART adults **(C)**, and deferred-cART adults **(D)**. cART, combination antiretroviral therapy.

Potential publication bias was assessed by funnel plot and Egger’s and Begg’s tests (**Figure 5**). Overall, the funnel plots were inverted symmetry, and no evidence of significant publication bias was obtained for the surveys that recruited early-treated children (**Figure 5A**, Egger’s test, *p* = 0.164; Begg’s test, *p* = 0.482), deferred-treated children (**Figure 5B**, Egger’s test, *p* = 0.421; Begg’s test, *p* = 0.111), early-treated adults (**Figure 5C**, Egger’s test, *p* = 0.571; Begg’s test, *p* = 0.144), or deferred-treated adults (**Figure 5D**, Egger’s test, *p* = 0.336; Begg’s test, *p* = 0.113).

### Association Between HIV-1 Reservoir Size and Anti-HIV-1 Antibody Levels

The publications about the association between anti-HIV-1 antibody response and HIV-1 reservoir size were identified through PubMed searches up to August 2021 using the following terms: “HIV persistence,” “latent reservoir,” “HIV reservoirs,” “cell associated HIV DNA,” “viral reservoirs,” “provirus burden,” “HIV serostatus,” and “HIV antibodies.” A total of 18 reports (17, 19, 21, 23, 27, 28, 32, 33, 38, 39, 57–64) were selected, and the median sample size of these studies was 48 (IQR: 22–98, N = 1,525). Out of 9 studies conducted in the children with vertical HIV-1 infection (17, 19, 21, 23, 27, 28, 32, 33, 62) with a median sample size of 30 (IQR: 23–83, N = 489), 6 (66.7%, 6/9) studies reported a significant association between anti-HIV titers and the size of HIV-1 reservoir (**Table 6**) (17, 19, 27, 28, 33, 62). Eight of the other 9 studies conducted in adult patients (median sample size = 51, IQR: 18–109, N = 1,106) also reported a significant association between HIV-1-specific antibody response and the reservoir size (**Table 7**) (39, 57–61, 63, 64). Of note, the majority of the studies to investigate the association between HIV-1 antibody response and HIV-1 reservoir size are in the HIV-infected subjects with viremia suppression (<50 copies/ml, **Tables 6** and **7**).

**Table 6:** Estimated statistical association between cell-associated HIV-1 DNA burden and quantitative or qualitative anti-HIV antibody in children with vertically HIV-1 infection.

Study ID (year, country)	Sample size	Median VL^§^ and age at cART initiation (months)	Median time on cART (months)	Viral load at antibody test	Median duration of virological suppression^†^ (months)	Antibody against HIV-1 proteins tested	Assays used to measure cell-associated HIV-1 DNA and antibody*	Main findings
Luzuriaga et al. **(2014, USA)** (17)	4	NA; 2.4	203.2	<20 copies/ml	NA	NA	dd-PCR	The cell-associated DNA level was lower in the seronegative group than in the seropositive group (*p* = 0.036).
	4	NA; 157.0	116.8				3rd-G EIA	
Persaud et al. **(2014, USA)** (19)	14	5.6; 2.4	150.9	<400 copies/ml	143.6	gp160, gp120, p66, p55, p51, gp41, p31, p24, and p17	dd-PCR	The median provirus burden for patients with a positive Western blotting profile was significantly larger than that in cases with indeterminate or negative HIV serostatus by Western blotting (*p* < 0.001).
	130	4.8; 32.9	124.1		87.6		WB	
Martínez-Bonet et al. **(2015, Spain)** (21)	23	5.6; 1.2	96.1	<200 copies/ml	54.8	gp120, gp41, p31, p24, p17	dd-PCR	No significant association was found between serological status and the cell-associated DNA level.
							3rd-G EIA	
Brice J et al. **(2017, Mali)** (23)	97	NA; 40.2	65.7	<50 copies/ml	NA	gp41	qPCR	No significant association was found between antibody response and the cell-associated DNA level.
							4th-G EIA	
Rocca et al. **(2019, Italy)** (27)	27	NA; 2.2	104.6	<50 copies/ml	68.1	gp160, gp120, p66, p55, p51, gp41, p39, p31, p24, p17	qPCR	Higher Western blotting band intensity score was correlated with higher total HIV-1 DNA levels (rho = 0.256, *p* = 0.032).
	42	NA; 42	165.5		66.9		WB	
McManus et al. **(2019, USA)** (62)	14	5.4; 1.8	NA	NA	NA	gp160	qPCR	Each unit increase in a log gp160 Ab was associated with a 6-fold increase in the odds of an HIV-1 DNA level >1,000 copies/10^6^ PBMCs (*p* < 0.001).
	8	4.8; 9.5					3rd-G EIA	
Bitnun et al. **(2020, Canada)** (28)	10	5.0; 2.4	120.5	<50 copies/ml	105.9	p24, gp41, gp120	qPCR	Quantitation of anti-HIV-1 antibody correlated directly with HIV-1 DNA level (rho = 0.34, *p* = 0.005).
	30	5.0; 38.9	152.1	<50 copies/ml	127.8		4th-G EIA	
	29	5.7; 82.7	124.1	<50 copies/ml	97.3			
Ajibola et al. **(2021, USA)** (32)	27	4.1; 0.03	19.5	74% <50 copies/ml	16.8	gp160, p24	dd-PCR	Cell-associated DNA values did not differ by serostatus.
							3rd-G EIA	
Veldsman et al. **(2021, South Africa)** (33)	30	3.8; 0	24.3	<10 copies/ml	NA	p24, gp41, gp120	qPCR	Cell-associated DNA values did differ by serostatus (*p* = 0.025).
							4th-G EIA	Antibody response correlated with HIV-1 DNA burden (rho = 0.84, *p* = 0.036).

NA, not available; dd-PCR, droplet digital PCR; qPCR, quantitative real-time PCR; PBMCs, peripheral blood mononuclear cells.

^§^Median pretreatment HIV-1 viral load (VL), log10 copies/ml.

^†^The definition of virological suppression varied among studies; viral load ranges from 20 to 400 copies/ml.

*2nd-G EIA, second-generation enzyme immunoassay; 3rd-G EIA, third-generation enzyme immunoassay; 4th-G EIA, fourth-generation enzyme immunoassay; WB, Western blotting.

**Table 7:** Estimated statistical association between cell-associated HIV-1 DNA burden and quantitative or qualitative anti-HIV antibody among adult cases.

Study ID (year, country)	Sample size	Median VL^§^ and months between diagnosis and cART initiation	Median time on cART (months)	Viral load at antibody test	Median duration of virological suppression^†^ (months)	Antibody against HIV-1 proteins tested	Assays used to measure cell-associated HIV-1 DNA and antibody*	Main findings
Burbelo et al. **(2014, USA)** (57)	10 EC^a^	—	—		—	P24, gp41, gp120, NC, RT, INT, PRO, Tat, matrix	qPCR	Compared to cART-treated subjects, both the total antibody titer and the integrated HIV-1 DNA burden were significantly lower in elite controllers (*p* = 0.036).
	9	4.4; NA	48.7	<50 copies/ml	NA		2nd-G EIA	
Lee et al. **(2016, USA)** (58)	51	4.7; 73	109.5	<40 copies/ml	36.5	p24, gp41, RT, INT, PRO, matrix	dd-PCR	Antibody responses to gp41 correlated with total HIV-1 DNA in PBMCs (rho = 0.48, *p* = 0.03).
							(20/51)	Antibody responses to PR correlated with total HIV-1 DNA in PBMCs (rho = 0.46, *p* = 0.04).
							2nd-G EIA	Antibody responses to matrix correlated with integrated HIV-1 DNA in resting CD4 T cells (rho = −0.79, *p* = 0.006).
							Alu-gag PCR	Antibody responses to PR correlated with total HIV-1 DNA in gut-associated lymphoid tissue (rho = 0.57, *p* = 0.020).
							(10/51)	
							2nd-G EIA	
							rtPCR	
							(16/51)	
							2nd-G EIA	
Keating et al. **(2017, USA)** (59)	31	NA; <6	60.8	<100 copies/ml	NA	p24, gp41	dd-PCR	Cell-associated DNA levels correlated with antibody response (rho = 0.25–0.30, *p* < 0.05).
	35	NA; >24				ENV	3rd-G EIA	
Stefic et al. **(2018, France)** (38)	44	5.3; 1.4	84.2	more than 90% <50 copies/ml	77.5	gp41	qPCR	Cell-associated HIV DNA level was not associated with serostatus.
							4th-G EIA	
Delagreverie et al. **(2019, France)** (60)	683	NA; 28	130.2	<50 copies/ml	80.3	gp41	qPCR	Cell-associated DNA levels correlated with antibody response (rho = 0.25, *p* = 0.0002).
							2nd-G EIA	
Keating et al. **(2019, USA)** (39)	101	4.6; >6	85.2	<50 copies/ml	74.0	p24, ENV	qPCR	Cell-associated DNA levels correlated with antibody response (rho = 0.35, *p* < 0.001).
							3rd-G EIA	
Giron et al. **(2019, Brazil)** (61)	116	NA; >6	NA	86.2% <100 copies/ml	68.1	gp41	qPCR	Patients with positive antibody quantitation showed slightly higher HIV total DNA (*p* = 0.09).
							2nd-G EIA	
Das et al. **(2020, USA)** (63)	17	NA; NA	NA	<40 copies/ml	NA	p24, gp41, gp120, gp160, RT, Tat, Vif, Nef	qPCR	Antibody responses to gp41 correlated with total HIV DNA (rho = 0.597, *p* = 0.011).
							2nd-G EIA	
Mastrangelo et al. **(2021, Italy)** (64)	9	NA; >6	NA	<50 copies/ml	121.7	gp120, PRO, INT	dd-PCR	Antibody against gp120 correlated with level of HIV DNA (rho = 0.70, *p* = 0.036).
							2nd-G EIA	Antibody against PRO correlated with level of HIV DNA (rho = 0.83, *p* = 0.005).
								Antibody against INT correlated with level of HIV DNA (rho = 0.66, *p* = 0.049).

NA, not available; NC, nucleocapsid; RT, reverse transcriptase; INT, integrase; PRO, protease; ENV, envelope; dd-PCR, droplet digital PCR; qPCR, quantitative real-time PCR; PBMCs, peripheral blood mononuclear cells.

a EC, elite controller, who is able to naturally control HIV-1 replication (viral load < 50 copies/ml) in the absence of therapy.

^§^Median pretreatment HIV-1 viral load (VL), log10 copies/ml.

^†^The definition of virological suppression varied among studies; viral load ranges from 20 to 400 copies/ml.

*2nd-G EIA, second-generation enzyme immunoassay; 3rd-G EIA, third-generation enzyme immunoassay; 4th-G EIA, fourth-generation enzyme immunoassay; WB, Western blotting.

Furthermore, several studies reported more cell-associated DNA copies in the HIV-1 seropositive population than the seronegative group (17, 33, 57, 61), and high levels of anti-HIV-1 binding antibodies positively correlated with HIV-1 DNA burden in peripheral blood mononuclear cells (PBMCs) with a Spearman’s correlation coefficient (rho) of 0.25~0.84 (*p* < 0.05) (27, 28, 33, 39, 58–60, 63, 64). However, inconsistent results were reported in other studies (21, 23, 32, 38). Stefic et al. reported no significant association between HIV-1 serostatus tested by HIV-1 rapid test and cell-associated HIV-1 DNA level in early-treated adults (38).

**Figure 6:**
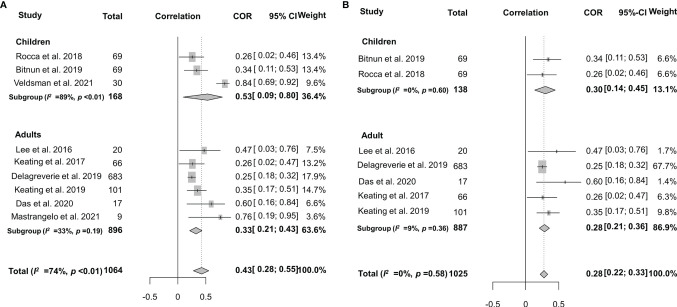
Forest plots of the pooled Spearman’s correlation coefficient (rho) with the corresponding 95% CIs for the correlation between HIV-1-specific antibody titer and HIV-1 DNA burden in patients for all the eligible studies **(A)** or the studies without significant heterogeneity **(B)**.

Spearman’s correlation coefficient between HIV-1-specific antibody titers and HIV-1 DNA burden was reported in 9 studies, although the heterogeneity was significant (*I* ^2^ = 74%, *p* < 0.01) (27, 28, 33, 39, 58–60, 63, 64). After adjusting intra-study and within-study correlations (**Figure 6**), we pooled the results together using random-effects meta-analyses and found a pooled rho of 0.43 (95% CI: 0.28–0.55) for all the studies analyzed. Further stratified analysis indicated that the pooled rho was 0.53 (95% CI: 0.09–0.80, *I* ^2^ = 89%, *p* < 0.01) for children and 0.33 (95% CI: 0.21–0.43, *I* ^2^ = 33%, *p* = 0.19) for adults (**Figure 6A**). In order to explore the source of heterogeneity, we conducted a sensitivity analysis and excluded two studies that were considered to be heterogeneous (33, 64). After the two heterogeneous studies were excluded, no notable heterogeneity was found in the studies included (*p* = 0.58). The overall pooled rho was 0.30 (0.14–0.45) and 0.28 (0.21–0.36) for children and adults, respectively (**Figure 6B**). Of note, Veldsman et al. reported a very strong correlation (rho = 0.84) between anti-HIV titers and reservoir size in children who received cART upon birth (33) probably due to the inhibition of both HIV-1 seeding from the reservoir and anti-HIV-1 antibody response during the very early cART (71, 76).

**Figure 7:**
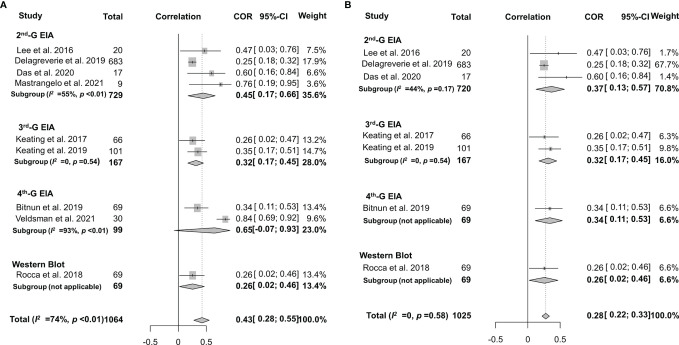
Forest plots of the pooled Spearman’s correlation coefficient (rho) with the corresponding 95% CIs for the correlation between HIV-1-specific antibody titer and HIV-1 DNA burden among HIV-infected population according to the serological test principles for all the eligible studies **(A)** or the studies without significant heterogeneity **(B)**.

**Figure 8:**
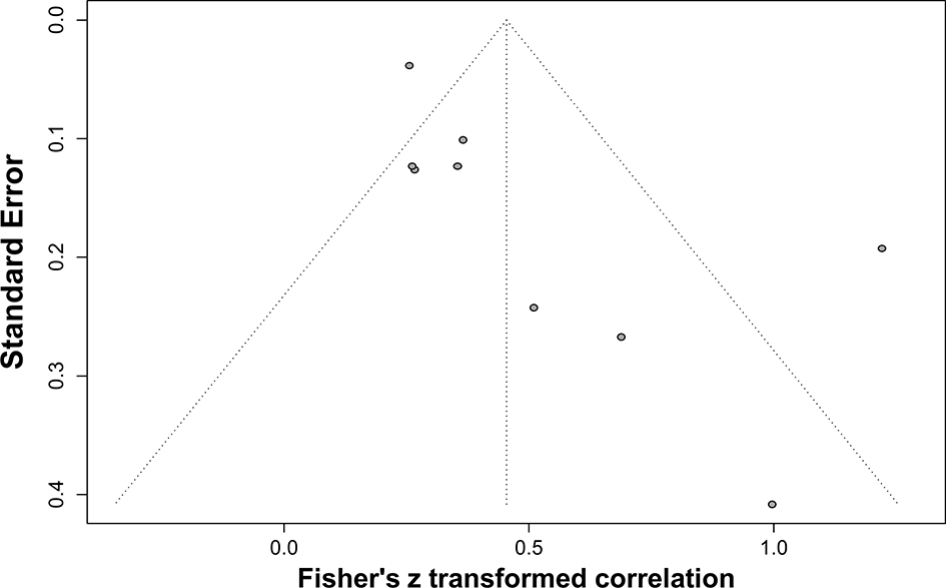
Funnel plot shows publication bias of the identified surveys to investigate the correlation between HIV-1-specific antibody titer and HIV-1 DNA burden.

As mentioned above, the frequency of seronegativity was significantly affected by the serological assays in particular HIV-1 rapid tests. Therefore, false-negative serostatus may underestimate the real association. To determine the potential impact of HIV-1 serological assays, we analyzed the correlation between anti-HIV antibody levels and the size of the latent reservoir under a stratified analysis according to the serological test principles (**Figure 7A**). We found that the 2nd-generation tests appeared to yield a slightly stronger correlation (rho = 0.37) than others (rho 0.26–0.34, **Figure 7B**). Potential publication bias was also assessed by funnel plot and Egger’s and Begg’s tests. The funnel plots were inverted symmetry, and no evidence of significant publication bias was obtained for the surveys (**Figure 8**, Egger’s test, *p* = 0.068; Begg’s test, *p* = 0.025).

## Discussion

Except for the window period of HIV-1 infection (77) or progression to AIDS (43, 78), HIV-1 seronegativity among cART-naïve patients is extremely rare (79). HIV-1 seronegativity caused by cART is a new issue that remains to be elucidated. In our systematic review, we uncover 38% and 16% of HIV-1 seronegativity in early-treated children and adults, respectively (**Table 3**). In contrast, only about 1% of deferred-treated children and adults were HIV-1 seronegative (**Table 3**). Furthermore, the 2nd generation of HIV-1 serological assays and rapid tests are prone to yield negative results for cART-treated patients (**Table 4**). To our best knowledge, this is the first systematic review to summarize the frequency of HIV-1 seronegativity in cART-treated patients. Negative HIV-1 serostatus caused by antiretroviral treatment may be due to the failure of seroconversion for the subjects who are acutely infected with HIV-1, or seroreversion caused by the efficient inhibition of HIV-1 replication, but does not mean the cure or eradication of HIV-1 infection since interruption of cART will result in virus rebound and rapid increase of anti-HIV antibody levels (75, 80–82). Since early cART would restrict HIV-1-specific antibody response and even result in seronegativity, the results of HIV-1 serological testing must be interpreted with caution in the era of cART.

Two key factors that may affect the frequency of HIV-1 seronegativity are the serological assays used to detect HIV-1 infection and the time to initiate cART. It is clear that the 2nd-generation EIA and rapid tests are more likely to result in seronegativity due to their relatively low sensitivity and specificity. The major difference between the 2nd- and 3rd-generation EIA is the detection of IgG antibodies against HIV-1 only for the 2nd-G EIA and both IgG and IgM antibodies for the 3rd-G EIA. O’Connell et al. found that the low sensitivity of HIV-1 rapid tests is due to its low efficiency of detecting antibodies against HIV-1 gp41, which is a major antibody produced in HIV-infected subjects (5). Therefore, the difference in seronegativity frequency detected by different generations of serological tests is because of the testing principles and the antigens used in the tests (36, 44). The difference in seronegativity reported in the studies may not be caused by sampling bias since the different performance of the 2nd-, 3rd-, and 4th-generation EIA and Western blotting analysis was reported when testing the same samples from an early-treated adult population (36). Compared to that in the 2nd-generation EIA, the frequency of HIV-1 seronegativity in the cART-treated children reduced by 22.0%, 27.0%, and 44.0% in the 3rd-G EIA, 4th-G EIA, and Western blotting, respectively (**Table 4**). For adult patients, the frequency of HIV-1 seronegativity reduced by 6.0%, 6.0%, and 3.0% in 3rd-G EIA, 4th-G EIA, and Western blotting, respectively (**Table 4**). These results clearly confirmed the better performance and reliability of the 4th-G EIA and Western blotting tests in detecting anti-HIV antibodies even in the era of cART. HIV-1 4th-G EIA can simultaneously detect IgM and IgG antibodies against HIV-1/-2 and group O virus as well as HIV-1 p24 antigen and shows superior sensitivity (83). It should thus be implemented as a routine assay when possible.

Furthermore, the time to initiate cART also significantly affects seronegativity among cART-treated patients. Early initiation of cART, in particular in children <6 months old or in adults with acute HIV-1 infection, will increase the probability of seronegativity. This is probably because early antiretroviral treatment inhibits the development of chronic HIV-1 infection, prevents maturation of specific immune responses against HIV-1, or restricts the size of HIV-1 reservoir (4, 7, 8, 12, 84–88). An excellent example is that early cART may help HIV-infected children to be cured or to achieve a functional cure to maintain HIV-1 free for a long time even in the absence of antiretroviral therapy (89–91). Moreover, it was reported that a higher frequency of HIV-1 seronegativity correlated well with a longer time of virological suppression (25, 38, 75). Time on virological suppression does not equal to, but theoretically should be proportional to, time on treatment. Taken together, early cART, long-term treatment, and efficient virus suppression may affect the immune response against HIV-1. At present, the best strategy to control HIV-1 epidemics is still early diagnosis, early initiation of antiretroviral therapy, and efficient viral suppression, which in turn may result in more seronegative cases. It is necessary to have a guideline to deal with the seronegative issue in the era of cART. HIV-1 serological assays should be evaluated in the serum samples from cART-treated subjects in order to further refine their design and improve their performance.

It is still unclear how antiretroviral therapy affects antibody response. Antibody generation and affinity maturation occur within germinal centers in lymph nodes where B cells mutate and are selected by T cells based on their ability to bind to an antigen (92). Moreover, cART can interfere with this process in multiple ways including reducing the production of HIV-1 antigens, which may be detrimental to antibody responses, but also halting or potentially reversing T-cell exhaustion and preventing CD4+ T cell decline, which may improve antibody responses. The obvious difference between the early and deferred-treated populations in antibody response may be due to the sufficient antigen harboring in the follicular dendritic cells to continuously stimulate antibody production in HIV-1 chronically infected subjects. Thus, a decline in the amount of circulating HIV-1 antigens after cART treatment would have little impact on the maintenance of antibody levels (93, 94). Also, it is well known that long-lived germinal centers in chronic HIV-1 infection can induce long-term affinity maturation of broadly neutralizing antibodies in response to viral escape mutants (95). However, different from the decreased HIV-1-specific binding antibody after antiretroviral treatment, there is no consensus on the role of antiretroviral therapy in the induction and maturation of anti-HIV-1 neutralizing antibodies (11, 48, 50–52, 54, 96–98) (**Table 8**). This discrepancy may also be partially caused by different cART regimens used (52), or different HIV-1 strains or inhibitory thresholds adopted in neutralization assay (11, 48).

**Table 8:** Effect of antiviral therapy on HIV-1-specific neutralizing antibodies (NAb) in adult patients.

Study ID (year, country)	Sample size	Therapy regimen	Therapy timing	Median time on cART (months)	Viral load (copies/ml)	HIV virus tested in Neutralization assay	NAb response
**Adherent treatment**							
Wainberg et al. **(1996, USA)** (52)	9	3TC	NA	NA	NA	HxB2; HxB2-MI84V; HxB2-K65R; HIV_IIIB_; two clinical isolates with MI84V mutation.	Titers of NAb rapidly decreased in AZT-treated individuals but stable in 3TC-treated patients.
	9	AZT	NA	NA	NA		
Sarmati et al. **(1997, Italy)** (54)	11	ZDV+SQV	Chronic	3.7	>100,000	Autologous isolates	No significant reduction of NAb titer.
	11	ZDV	Chronic	3.7	>100,000	Autologous isolates	Significant reduction in 5 patients.
	11	SQV	Chronic	3.7	>100,000	Autologous isolates	Significant reduction in 2 subjects.
Dreyer et al. **(1999, USA)** (48)	24	Two NRTI or NNRTIs plus PI	NA	NA	<400	HIV-1 NL4-3, HIV-1 NFN-SX, HIV-1 BaL viruses and 6 primary isolates: P59423, W25798, W79290, V89872, V67970, W179273	Use of antiviral drugs contributed to development of broadly NAb.
Binley et al. **(2000, USA)** (11)	7	Two NRTI or NNRTIs plus PI	Acute	NA	<400	Autologous isolates	2 patients without NAb response.
	11		Chronic		<400	Autologous isolates	No NAb response in all subjects.
Kim et al. **(2001, USA)** (96)	11	Two NRTI or NNRTIs plus PI	Chronic	NA	<400	Three primary HIV-1 isolates (BZ167, US1, and CM237)	NAb titers increased after treatment.
Sarmati et al. **(2001, Italy)** (97)	33	ZDV+3TC	Chronic	NA	>15,000	Autologous isolates	NAb activity was detected in all patients.
Falkensammer et al. **(2007, Austria)** (51)	10	Two NRTI or NNRTIs plus PI	Chronic	NA	<100	HIV_IIIB_	NAb titer decreased in 7 subjects.
Medina-Ramírez et al. **(2011, Spain)** (50)	173	Two NRTI or NNRTIs plus PI	Chronic	60.8	<50	6 recombinant viruses	Broadly neutralizing activity found in 1.7% (3/174) patients.
Gach et al. **(2014, USA)** (98)	51	Two NRTI or NNRTIs plus PI	Chronic	NA	<50	Pseudotyped viruses (HIV-1JR-FL) and replication competent viruses (HIV-2/HIV-1 chimeras)	HIV-1JR-FL and HIV-2/HIV-1 MPER chimera can be neutralized in 11 and 4 subjects, respectively.

NA, not available; 3TC, lamivudine; AZT/ZDV, zidovudine; SQV, saquinavir; NRTI, nucleotide reverse transcriptase inhibitor; NNRTI, non-nucleotide reverse transcriptase inhibitor; PI, protease inhibitor; STI, structured treatment interruption; ATI, analytic treatment interruption.

Furthermore, Zaongo et al. have recently reviewed the role of cART and HIV-1 VL in influencing HIV-1-specific antibody response (99). The HIV-1-specific antibody is usually detected 14 days after infection in adults (100); however, HIV-infected infants develop autogenous antibodies approximately at the age of 4 months (101). Therefore, the window period for cART to impede the autogenous antibody response against HIV-1 is much longer in infants than in adults. These findings may explain the higher frequency of HIV-1 seronegativity in early-treated children (38.0%) than in early-treated adults (16.0%).

The correlation between HIV-1 VL and antibody response is another debated issue. Unfortunately, HIV-1 VLs vary among HIV-infected subjects and are affected by HIV-1 subtypes (102, 103). In this meta-analysis, only 3 (10.7%, 3/28) studies (21, 35, 38) provided information on HIV-1 subtypes as a whole, but not for every HIV-infected individual in these studies, and it is impossible to analyze the correlation between HIV-1 subtypes and seronegativity. However, when pretreatment HIV-1 RNA levels were included in the meta-regression model, the results showed that pretreatment HIV-1 VL did not significantly affect the frequency of seronegativity (**Table 5**). Furthermore, it is more likely that higher VLs result in both larger reservoirs and more strong antibody responses; however, small HIV-1 reservoirs and a low level of anti-HIV antibodies were observed in cART-treated patients who are under virological suppression, especially for early-treated populations (19, 84, 104). Therefore, based on their synchronous response to cART, it is reasonable to assume that anti-HIV antibody levels may be a suitable proxy for the size of the latent reservoir. Our meta-analysis results indicated that the levels of anti-HIV-1 antibody response were positively associated with the size of HIV-1 reservoir and supported that anti-HIV titers might be a feasible biomarker of HIV-1 reservoir burden and may be used to assess the efficiency of cART in particular in the resource-limiting settings, although further studies are needed (99).

The major limitation of our analysis is the missing data for some important factors in the majority of the studies, including HIV-1 subtypes, pretreatment CD4+ cell counts, and ethnicity (84, 99). Because only 10.7% (3/28) and 11.1% (2/18) studies reported the HIV-1 subtype and ethnicity/race of study subjects, we did not analyze the effect of viral subtypes and ethnicity in cART-induced seronegativity or HIV-1 reservoir. The independent effect of these factors in antibody response or HIV-1 reservoir size should be further assessed.

In conclusion, seronegativity in the HIV-infected and cART-treated populations is an important issue particularly when cART is initiated during acute/early HIV-1 infection. In the era of cART, it is definitely necessary to develop an appropriate algorithm for HIV-1 diagnosis and management of cART-treated individuals. Additionally, more studies are needed for further validation of the HIV-1-specific antibody as a feasible biomarker of the HIV-1 reservoir.

## Data Availability Statement

The original contributions presented in the study are included in the article/**Supplementary Material**, further inquiries can be directed to the corresponding author.

## Author Contributions

ST and YL conceived the study. YL and HL collected, analyzed, and interpreted the data. YL made the tables and figures and wrote the manuscript. ED and ST revised the manuscript. All authors reviewed, revised, and approved the final report.

## Funding

This work was supported by the National Major Science and Technology Project (grant number 2018ZX10732-401-003-003).

## Conflict of Interest

The authors declare that the research was conducted in the absence of any commercial or financial relationships that could be construed as a potential conflict of interest.

## Publisher’s Note

All claims expressed in this article are solely those of the authors and do not necessarily represent those of their affiliated organizations, or those of the publisher, the editors and the reviewers. Any product that may be evaluated in this article, or claim that may be made by its manufacturer, is not guaranteed or endorsed by the publisher.
