# Supplementary material for: Influence of Combination Antiretroviral Therapy on HIV-1 Serological Responses and Their Implications: A Systematic Review and Meta-Analysis
Source: Front Immunol. 2022 Mar 30;13:844023. doi: 10.3389/fimmu.2022.844023 (PMC9006953; doi:10.3389/fimmu.2022.844023)
Supplement: Supplementary file 1 [file DataSheet_1.pdf]

Checklist S1. Checklist of items to include when reporting a systematic review or meta-analysis

Section/topic	#	Checklist item	Reported paragraph # (within section)
**TITLE**			
Title	1	Identify the report as a systematic review, meta-analysis, or both.	Both
**ABSTRACT**			
Structured summary	2	Provide a structured summary including, as applicable: background; objectives; data sources; study eligibility criteria, participants, and interventions; study appraisal and synthesis methods; results; limitations; conclusions and implications of key findings; systematic review registration number.	Abstract was structured in format required by Journal
**INTRODUCTION**			
Rationale	3	Describe the rationale for the review in the context of what is already known.	1-2
Objectives	4	Provide an explicit statement of questions being addressed with reference to participants, interventions, comparisons, outcomes, and study design (PICOS).	2
**METHODS**			
Protocol and registration	5	Indicate if a review protocol exists, if and where it can be accessed (e.g., Web address), and, if available, provide registration information including registration number.	N/A
Eligibility criteria	6	Specify study characteristics (e.g., PICOS, length of follow-up) and report characteristics (e.g., years considered, language, publication status) used as criteria for eligibility, giving rationale.	1
Information sources	7	Describe all information sources (e.g., databases with dates of coverage, contact with study authors to identify additional studies) in the search and date last searched.	1

Section/topic	#	Checklist item	Reported paragraph # (within section)
Search	8	Present full electronic search strategy for at least one database, including any limits used, such that it could be repeated.	1, Appendices
Study selection	9	State the process for selecting studies (i.e., screening, eligibility, included in systematic review, and, if applicable, included in the meta-analysis).	2
Data collection process	10	Describe method of data extraction from reports (e.g., piloted forms, independently, in duplicate) and any processes for obtaining and confirming data from investigators.	3
Data items	11	List and define all variables for which data were sought (e.g., PICOS, funding sources) and any assumptions and simplifications made.	3
Risk of bias in individual studies	12	Describe methods used for assessing risk of bias of individual studies (including specification of whether this was done at the study or outcome level), and how this information is to be used in any data synthesis.	4
Summary measures	13	State the principal summary measures (e.g., risk ratio, difference in means).	4
Synthesis of results	14	Describe the methods of handling data and combining results of studies, if done, including measures of consistency (e.g., I^2) for each meta-analysis.	4
Risk of bias across studies	15	Specify any assessment of risk of bias that may affect the cumulative evidence (e.g., publication bias, selective reporting within studies).	4
Additional analyses	16	Describe methods of additional analyses (e.g., sensitivity or subgroup analyses, meta-regression), if done, indicating which were pre-specified.	4
**RESULTS**			
Study selection	1	Give numbers of studies screened, assessed for eligibility, and included in the review, with reasons for	1, Fig. 1

Section/topic	#	Checklist item	Reported paragraph # (within section)
	7	exclusions at each stage, ideally with a flow diagram.	
Study characteristics	18	For each study, present characteristics for which data were extracted (e.g., study size, PICOS, follow-up period) and provide the citations.	1, 2, Table 1 and 2
Risk of bias within studies	19	Present data on risk of bias of each study and, if available, any outcome-level assessment (see Item 12).	N/A
Results of individual studies	20	For all outcomes considered (benefits or harms), present, for each study: (a) simple summary data for each intervention group and (b) effect estimates and confidence intervals, ideally with a forest plot.	Fig. 2, Fig.3, Fig. 4, Fig. 5
Synthesis of results	21	Present results of each meta-analysis done, including confidence intervals and measures of consistency.	Fig. 2, Fig.3, Fig. 4, Fig. 5
Risk of bias across studies	22	Present results of any assessment of risk of bias across studies (see Item 15).	6
Additional analysis	23	Give results of additional analyses, if done (e.g., sensitivity or subgroup analyses, meta-regression [see Item 16]).	5
**DISCUSSION**			
Summary of evidence	24	Summarize the main findings including the strength of evidence for each main outcome; consider their relevance to key groups (e.g., health care providers, users, and policy makers).	1-2
Limitations	25	Discuss limitations at study and outcome level (e.g., risk of bias), and at review level (e.g., incomplete retrieval of identified research, reporting bias).	1-2
Conclusions	26	Provide a general interpretation of the results in the context of other evidence, and implications for future research.	1-2

Section/topic	#	Checklist item	Reported paragraph # (within section)
**FUNDING**			
Funding	27	Describe sources of funding for the systematic review and other support (e.g., supply of data); role of funders for the systematic review.	See Funding in manuscript

Appendices for details of the search strategy for databases

Appendix 1. MEDLINE (PubMed) search strategy queries

Date range: earliest index date to 08/2021

HIV/AIDS terms

#1 “HIV Infections” [MeSH]

#2 “HIV” [MeSH]

#3 hiv[tw]

#4 hiv-1*[tw]

#5 hiv-2*[tw]

#6 hiv1[tw]

#7 hiv2[tw]

#8 hiv infect*[tw]

#9 human immunodeficiency virus[tw]

#10 human immunedeficiency virus[tw]

#11 human immuno-deficiency virus[tw]

#12 human immune-deficiency virus[tw]

#13 ((human immun*) AND (deficiency virus[tw]))

#14 acquired immunodeficiency syndrome[tw]

#15 acquired immunedeficiency syndrome[tw]

#16 acquired immuno-deficiency syndrome[tw]

#17 acquired immune-deficiency syndrome[tw]

#18 ((acquired immun*) AND (deficiency syndrome[tw]))

#19 #1 OR #2 OR #3 OR #4 OR #5 OR #6 OR #7OR #8 OR #9 #10 #11 OR #12 OR #13 OR #14 OR #14 OR #15 OR #16 OR #17OR #18

Therapy terms

#20 anti-retroviral agents/therapeutic use [Mesh Terms]

#21 anti-HIV agents [MeSH Terms]

#22 antiretroviral therapy, highly active [MeSH Terms]

#23 antiretroviral therapy[tw]

#24 antiretroviral*[tw]

#25 anti-retroviral[tw]

#26 HAART [tw]

#27 cART [tw]

#28 ART [tw]

#29 ARV*[tw]

#30 Pre-exposure prophylaxis[tw]

#31 #21 OR #22 OR #23 OR #24 OR #25 OR #26 OR #27OR #28 OR #29 OR #30

Performance trems

#32 specificity [MeSH Terms]

#33 sensitivity [MeSH Terms]

#34 reactivity [tw]

#35 accuracy [tw]

#36 reliability [tw]

#37 false negativity[tw]

#38 false positivity[tw]

#39 seropositivity [tw]

#40 seronegativity [tw]

#41 seroreversion[tw]

#42 seroconversion [MeSH Terms]

#43 performance[tw]

#44 #32 OR #33 OR #34 OR #35 OR #36 OR #37 OR #38 OR #39 OR #40 OR #41 OR #42 OR #43

Serological test terms

#45 serology [MeSH Terms]

#46 serological test[tw]

#47 diagnositic test[tw]

#48 serological diagnosis[tw]

#49 antibody test[tw]

#50 immunoassay [tw]

#51 rapid antibody test [tw] OR rapid test [tw] OR point-of-care immunoassays [tw] OR point-of-care test [tw]

#52 enzyme immunoassay [tw]

#53 enzyme linked immunosorbent assay [tw] OR ELISA [tw]

#54 immunoblot [tw] OR western blot [tw] OR WB [tw]

#55 #45 OR #46 OR #47 OR #48 OR #49 OR #50 OR #51 OR #52 OR #53 OR #54

Terms combination

#56 #19 AND #31 AND #44 AND #55

Appendix 2. search strategy queries in EMBASE and Cochrane Library

Date range: earliest index date to 08/2021

('hiv' OR 'human immunodeficiency virus' OR 'aids' OR 'acquired immunedeficiency syndrome') AND ('sensitivity' OR 'specificity' OR 'reactivity' OR 'nonreactivity' OR 'accuracy' OR 'negativity' OR 'positivity' OR 'performance' OR 'seroreversion') AND ('diagnosis' OR 'antibody test' OR 'immunoassay' OR 'ia' OR 'rapid antibody test' OR 'rapid test' OR 'point-of-care immunoassays' OR 'point-of-care test' OR 'poc' OR 'enzyme immunoassay' OR 'eia' OR 'enzyme linked immunosorbent assay' OR 'elisa' OR 'immunoblot' OR 'immuno-blot' OR 'western blot' OR 'western-blot' OR 'wb') AND ('haart' OR 'art' OR 'cart' OR 'arv' OR 'antiretroviral therapy' OR 'highly active antiretroviral therapy')

Appendix 3. search strategy queries in Web of Science

Date range: earliest index date to 08/2021

#1 "HIV Infections" OR "HIV" OR hiv OR hiv-1* OR hiv-2* OR hiv1 OR hiv2 OR hiv infect* OR human immunodeficiency virus OR human immunedeficiency virus OR human immuno-deficiency virus OR human immune-deficiency virus OR ((human immun*) AND (deficiency virus)) OR acquired immunodeficiency syndrome OR acquired immunedeficiency syndrome OR acquired immuno-deficiency syndrome OR acquired immune-deficiency syndrome OR ((acquired immun*) AND (deficiency syndrome))

#2 anti-retroviral agents OR therapeutic use OR anti-HIV agents OR antiretroviral therapy, highly active OR antiretroviral therapy OR antiretroviral* OR anti-retroviral OR HAART OR cART OR ART OR ARV* OR Pre-exposure prophylaxis

#3 specificity OR sensitivity OR reactivity OR accuracy OR reliability OR false negativity OR false positivity OR seropositivity OR seronegativity OR seroreversion OR seroconversion OR performance

#4 serology OR serological test OR diagnositic test OR serological diagnosis OR antibody test OR immunoassay OR rapid antibody test OR rapid test OR point-of-care immunoassays OR point-of-care test OR enzyme immunoassay OR enzyme linked immunosorbent assay OR ELISA OR immunoblot OR western blot OR WB

#5 #1 AND # 2 AND # 3 AND # 4
